# The Respiration in Pressure on the Brain

**Published:** 1853-03

**Authors:** 


*The Respiration in Pressure on the Brain.* By Dr. LANDGRAF.—Dr. Landgraf calls attention to the state of the respiration, in cases of cerebral pressure. It is frequently not stertorous and labored, as described in books, till the agony; but it is interrupted; that is to say, after from six to twelve tranquil and easy respirations, a long pause insues. The author details cases in proof of the existence and diagnostic value of this sign.—*Ibid., from Deutsche Klinik,* 1852, p. 39.

